# 5-Fluorouracil—Complete Insight into Its Neutral and Ionised Forms

**DOI:** 10.3390/molecules24203683

**Published:** 2019-10-13

**Authors:** Justyna Wielińska, Andrzej Nowacki, Beata Liberek

**Affiliations:** Faculty of Chemistry, University of Gdańsk, Wita Stwosza 63, 80-308 Gdańsk, Poland

**Keywords:** 5-Fluorouracil, protonation, deprotonation, charge delocalisation, p*K*_a_ value, DFT methods

## Abstract

5-Fluorouracil (5FU), a common anti-cancer drug, occurs in four tautomeric forms and possesses two potential sites of both protonation and deprotonation. Tautomeric and resonance structures of the ionized forms of 5FU create the systems of connected equilibriums. Since there are contradictory reports on the ionized forms of 5FU in the literature, complex theoretical studies on neutral, protonated and deprotonated forms of 5FU, based on the broad spectrum of DFT methods, are presented. These indicate that the O4 oxygen is more willingly protonated than the O2 oxygen and the N1 nitrogen is more willingly deprotonated than the N3 nitrogen in a gas phase. Such preferences are due to advantageous charge delocalization of the respective ions, which is demonstrated by the NBO and ESP analyses. In an aqueous phase, stability differences between respective protonated and deprotonated forms of 5FU are significantly diminished due to the competition between the mesomeric effect and solvation. The calculated p*K*_a_ values of the protonated, neutral and singly deprotonated 5FU indicate that 5FU does not exist in the protonated and double-deprotonated forms in the pH range of 0–14. The neutral form dominates below pH 8 and the N1 deprotonated form dominates above pH 8.

## 1. Introduction

The anti-cancer drugs are a widely applied group of pharmaceuticals. These are hazardous to humans due to their cytotoxicity, genotoxicity, mutagenicity and teratogenicity. Extensively consumed anti-cancer drugs are detected in the environment in increasing amounts, where they are hazardous to wildlife [1,2]. Among anti-cancer drugs, 5-fluorouracil (5FU) is one of the most commonly used [3]. It is an analogue of natural thymine and has been proven useful in the chemotherapy of different cancers [4]. 5FU is also known to act as an antifungal drug [5].

5-Fluorouracil is a weak acid with a measured p*K*_a_ of 7.93 [6] or 8.05 [7]. This means that 5FU is, to a great extent, deprotonated at physiological and intracellular pH, as well as at environmental pH, particularly when it is slightly basic (some soils, seawater). The ionized form of 5FU influences its mobility in the body fluid [8] and in the environment [9]. Interaction with the proteins [10] and crossing the cell membranes [11,12,13] also depend on the form (ionized or neutral) of 5FU. In the case of tumours, the ability of drugs to cross the cell membrane is strongly dependent on the plasmalemmal pH gradient [14,15]. It has been demonstrated that the tumour extracellular environment is acidic, whereas the intracellular environment is neutral to alkaline [16]. According to the Henderson–Hasselbalch equation, different pH outside and inside of a tumor cell means different concentrations of the neutral and deprotonated forms of an acidic drug in these places. Therefore, it is crucial to know how the deprotonated form of 5FU looks. It was demonstrated that the form of 5FU influences the rate of its removal via photocatalytic oxidation in an aqueous environment [17]. It was also demonstrated that the ionized form of 5FU is primarily responsible for 5FU-induced mutagenesis [18]. No wonder that the acidic properties of 5FU are a subject of permanent studies, both experimental, aimed at precise p*K*_a_ measurements, and theoretical, in an attempt to indicate the site of 5FU deprotonation. Since 5FU possesses two potential sites of deprotonation, on the amide N1 and N3 nitrogens (for the atoms numbering system, see Schemes 3 or 4), there is ongoing discussion of the preferable deprotonated form of 5FU in the literature. This discussion has not unambiguously settled the problem so far. With reference to the gas phase, it is rather commonly accepted that the N1 anionic form of 5FU is more stable than the N3 anionic form [19,20,21]. However, the recent report of Mioduszewska et al. [22] ascribed the measured p*K*_a1_ value of 7.5 to the N3-H species, based on the atomic charges calculated in the gas phase. Large discrepancies in the assignment of protonated and deprotonated forms of 5FU are particularly seen when calculations are provided in the aqueous phase. Some calculations demonstrate that deprotonation from the N3 nitrogen atom is more favorable than from the N1 nitrogen [19]; however, others indicate the opposite preference [21]. The results based on the Raman spectra of 5FU supported by DFT optimizations [23,24] or on the NMR data [25] are also ambiguous. These make the site of deprotonation in 5FU depend on pH, whereas the extent of deprotonation solely depends on pH, not the site. Importantly, the second p*K*_a2_ value of 5FU remains uncertain. There are two different values of p*K*_a2_ 9 [22] and p*K*_a2_ 13 [26] found in the literature for 5FU.

Because of indicated discrepancies in the ascribing of ionized forms of 5FU in the literature, new and complete insight into neutral, protonated and deprotonated forms of 5FU, based on the broad spectrum of DFT methods, in the gas and aqueous phases, are presented herein. For the first time, the protonated forms of 5FU in water are reported and, also for the first time, the NBO and ESP analyses for ionized forms of 5FU are presented. Additionally, the p*K*_a_ values of the protonated, neutral and singly deprotonated 5FU are calculated. Based on these data, it is possible to predict the form of 5FU at any pH. 

## 2. Methodology

### 2.1. Geometry Optimisations

All of the calculated structures of 5FU were prepared in the MOLDEN program [27], followed by optimisations using Gaussian 09 [28]. Optimisation of the structures was performed using four functionals: A Becke’s three-parameter hybrid exchange functional [29] involving the gradient-corrected correlation functional of Lee, Yang and Parr (B3LYP) [30], the two Truhlars’ functionals (M052X [31] and M062X [32]) and the WB97XD functional [33] combined with the 6-31+G**, 6-311++G** and aug-cc-pVDZ basis sets in the gas phase. In some cases, CBS-QB3 calculations [34] were conducted in order to have a reference point for DFT methods. The tight option was used for SCF convergence.

The frequency calculations performed on these geometries at the same level of theory ensured that all optimised structures are true energy minima on the potential energy surface with no imaginary frequency. Furthermore, frequency calculations to a true energetic minimum on the potential energy surface allowed the unscaled zero-point (ZPVE) and thermal energy corrections required to assess the Gibbs free energy of all the forms in the gas phase to be obtained. 

Next, all of the gas-phase geometries were optimised in aqueous solution using the PCM [35] and SMD [36] solvation models, respectively. PCM is one of the oldest solvation models successfully used for the p*K*_a_ calculations [37,38,39,40]. SMD is a newer solvation model, which turned out to be suitable to any charged or uncharged solute in any solvent or liquid medium [41]. The alpha scaling factors of 1.0 and 1.2 were used in the calculations.

As a result of geometry optimisation, the total electronic energies, *E*_tot_, were obtained. Then, the thermochemical analysis was performed based on the harmonic vibrational frequencies. In this way, the zero-point energy, ZPE, and thermal correction to the energy, E_(0-298)_, were obtained. The sum of the total energy (*E*_tot_) and the ZPE gave the zero-point-corrected total energy, *E*_0_. Calculation of the enthalpy at 298.15 K was based on the equation *H*_298_ = *E*_298_ + *RT*, where *E*_298_ is the sum of the electronic energy and the thermal correction to the energy (*E*_0_ + *E*_(0-298)_). Calculation of the Gibbs free energy (sum of the electronic and thermal free energies) at 298.15 K was based on the equation *G*_298_ = *H*_298_ − *TS*_298_.

The atomic charge distribution was determined by fitting to the electrostatic potential at points selected according to the Kollman’s scheme (ESP) for the optimised anions [42]. Additionally, the natural bond orbital (NBO) analysis was carried out for the optimised ions using version 3.1 of the NBO package [43] included in the Gaussian 09 program at the B3LYP/6-311++G** and M062X/6-311++G** levels of theory in the gas phase.

### 2.2. Methods of the pK_a_ Evaluations 

In order to calculate the p*K*_a_ values of 5FU, two methodologies were applied, i.e., the direct and relative method, both with PCM and SMD solvation models, respectively.

**Scheme 1:**
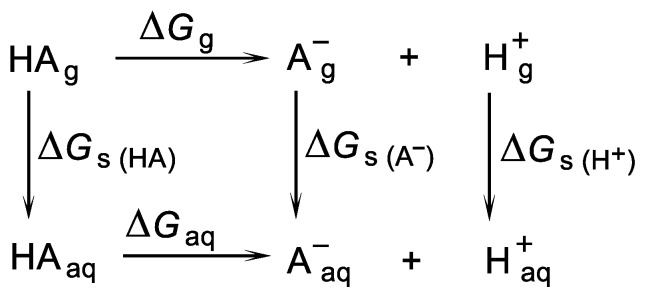
A thermodynamic cycle in which an acid species HA is dissociated into its conjugated base A^−^ and a proton.

The direct method is the most common and, conceptually, the simplest method of p*K*_a_ calculation, which is based on the process of abstraction of the proton from the acid to form the conjugate base A^−^ and H^+^ [37,40,44,45,46,47,48] This method involves a thermodynamic cycle that combines the gas-phase Gibbs free energy of deprotonation (Δ*G*_g_) and the solvation energies (Δ*G*_s_) of all particles (Scheme 1).

The Gibbs free energy change of HA deprotonation in aqua (Δ*G*_aq_) was calculated according to Equation (1):

Δ*G*_aq_ = Δ*G*_g_ + ΔΔ*G*_s_ + 1.89 kcal/mol (1)

where Δ*G*_g_ as the change of Gibbs free energy of HA deprotonation in a gas phase is calculated according to Equation (2):

Δ*G*_g_ = *G* (A^−^)_g_ + *G* (H^+^)_g_ − *G* (HA)_g_ (2)

whereas ΔΔ*G*_s_ is defined in Equation (3):

ΔΔ*G*_s_ = Δ*G*_s_ (A^−^) + Δ*G*_s_ (H^+^) − Δ*G*_s_ (HA) (3)

where Δ*G*_s_ (HA), Δ*G*_s_ (A^−^) and Δ*G*_s_ (H^+^) are the changes of Gibbs free energies of HA, A^−^ and H^+^ hydrations.

Since the calculation of ∆*G*_g_ uses a reference standard state of 1 atm and the calculations of ∆*G*_s_ use a reference standard state of 1 M concentration, converting the ∆*G*_g_ reference state from 1 atm to 1 M is accomplished using Equations (4) and (5) [49]:

∆*G*_g_ (1 M) = ∆G_g_ (1 atm) + *RT* ln (24.46) (4)

*RT* ln 24.4654 = 1.89 kcal/mol (5)

The values of −6.28 kcal/mol for G (H^+^) _g_ and −265.9 kcal/mol for ∆G_s_ (H^+^) were derived from the literature [50,51].

Having Δ*G*_aq_, a required p*K*_a_ value can be calculated using Equation (6):

(6) $$pK_{a}=\frac{\Delta G_{aq}}{2.303RT}$$

**Scheme 2:**
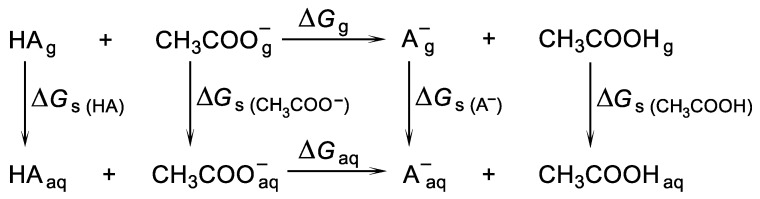
Thermodynamic cycle in which an acid HA donates a proton to CH_3_COO^−^ to yield the conjugated base A^−^ and CH_3_COOH.

The relative (also known as an isodesmic) [52] method we used is based on the process in which acid HA donates a proton to the selected base (here acetate ion) to yield its conjugated base A^−^ and another acid, conjugated with the base used (here, acetic acid). This method involves a thermodynamic cycle, in which the same number of molecules is found on both sides of the reaction equation (Scheme 2). Such a situation gives an advantage over the cycle used in the direct method since solvation energy errors are largely cancelled out, which should improve the accuracy of the p*K*_a_ predictions [53,54,55,56]. This cancellation of errors is expected to be maximised when the molecular and electronic structures of acids and conjugated bases are similar.

In the relative method, the p*K*_a_ of the acid considered is expressed in terms of the acidity of another acid, for which the experimental p*K*_a_ is known (here, acetic acid), according to Equation (7):

(7) $$pK_{a}=\frac{\Delta G_{aq}}{2.303RT}+pK_{a}CH_{3}COOH$$

The p*K*_a_ value of 4.76 was used for CH_3_COOH [57].

The same two methodologies, based on Equation (8) (direct method) and Equation (9) (relative method), were used in the case of protonated 5FU.

HA^+^ → A + H^+^ (8)

HA^+^ + CH_3_COO^−^ → A + CH_3_COOH (9)

## 3. Results and Discussion

### 3.1. Relationship between Tautomeric, Deprotonated and Protonated forms of 5FU

**Scheme 3:**
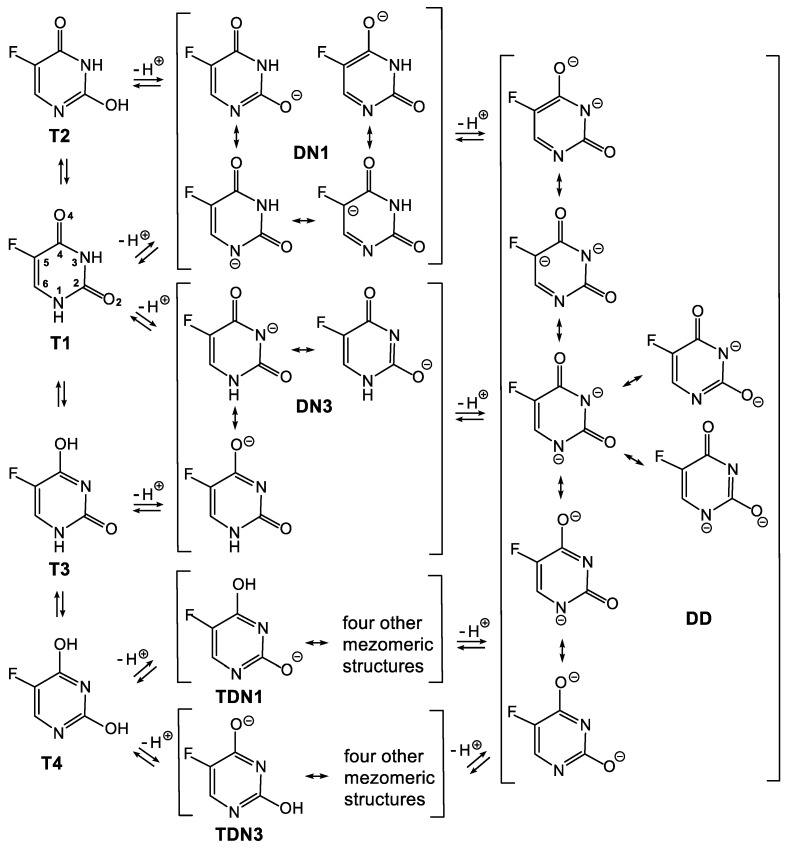
System of connected equilibriums between tautomeric and deprotonated forms of 5FU. The latter are described by respective resonance structures. T1–T4—tautomeric forms; DN1 and DN3—forms deprotonated at the N1 and N3, respectively; DD—double-deprotonated form; TDN1 and TDN3—tautomeric forms of DN1 and DN3, respectively.

**Scheme 4:**
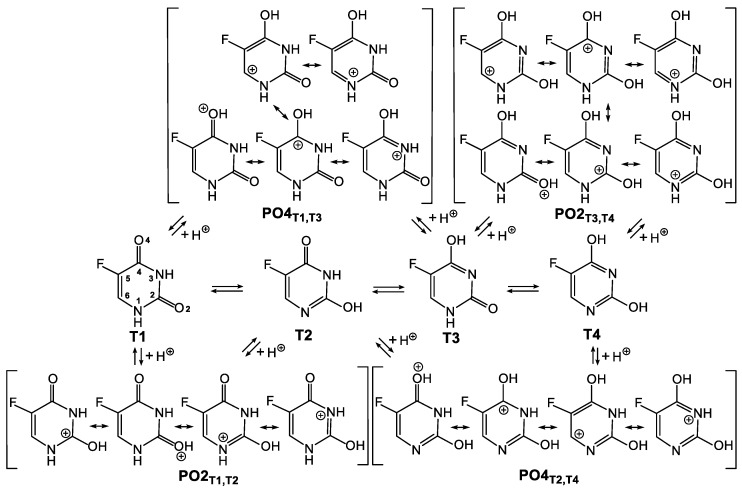
System of connected equilibriums between tautomeric and protonated forms of 5FU. The latter are described by respective resonance structures. T1–T4—tautomeric forms; PO2_T1,T2_—form protonated at the O2, derived from tautomeric forms T1 or T2; PO2_T3,T4_—form protonated at the O2, derived from tautomeric forms T3 or T4; PO4_T1,T3_—form protonated at the O4, derived from tautomeric forms T1 or T3; PO4_T2,T4_—form protonated at the O4, derived from tautomeric forms T2 or T4.

5-Fluorouracil is a compound which not only possesses two potential sites of deprotonation, but also occurs in four tautomeric forms (T1, T2, T3 and T4 presented in Scheme 3 and Scheme 4, respectively). Additionally, each of the deprotonated forms of 5FU is described by a few resonance structures. Moreover, deprotonation of two different tautomeric forms of 5FU may provide the same deprotonated species. Thus, when the N1-H is deprotonated from the tautomeric form T1 and when the O2-H is deprotonated from the tautomeric form T2, the same deprotonated species DN1 is formed (Scheme 3). Analogously, when the N2-H is deprotonated from the tautomeric form T1 and when the O4-H is deprotonated from the tautomeric form T3, the same deprotonated species, DN3, is formed. In turn, deprotonation of the tautomeric form T4 provides tautomeric forms of DN1 and DN3, respectively. The succeeding deprotonation of 5FU provides one dianion (DD), irrespective of the starting tautomer and singly deprotonated species.

The situation is similarly complicated in the case of 5FU protonation, where starting from different tautomers, one may achieve the same protonated form. Thus, protonation of the both T1 and T2 tautomeric forms may provide the same PO2_T1,T2_ species, whereas protonation of the both T1 and T3 tautomeric forms may provide the same PO4_T1,T3_ species (Scheme 4). Analogously, protonation of the both T3 and T4 tautomeric forms may provide the same PO2_T3,T4_ species, whereas protonation of the both T2 and T4 tautomeric forms may provide the same PO4_T2,T4_ species.

Presented systems of the connected equilibriums show the complexity of the theoretical considerations on acidity and basicity of 5FU.

### 3.2. Stability of the Tautomeric Forms of 5FU

**Figure 1:**
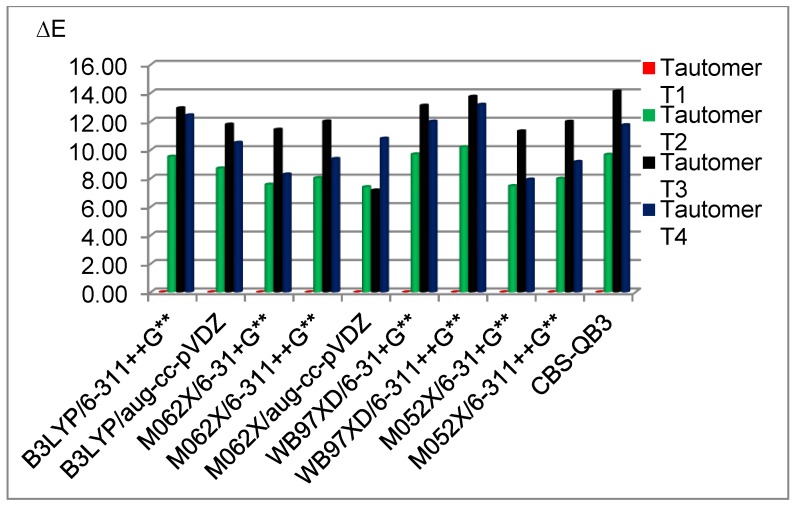
The relative energies of 5FU tautomers at different computational methods in the gas phase. Energies are referred to as the most stable tautomer T1.

At the beginning, geometry optimisations of the four tautomeric forms of 5FU were performed using a broad spectrum of DFT methods and one CBS-QB3 method. Our results show the advantaged stability of the T1 tautomer, which is 7.32–10.38 kcal/mol (gas phase) or 6.48–11.68 kcal/mol (water) more stable than the second most stable T2 tautomer (Table 1). Tautomer T3 is the least stable in a gas phase with Δ*G*_T_ in the range of 10.88–13.82 kcal/mol, depending on the method used (Table 1). In turn, the T4 form is the least stable in water with Δ*G*_T_ in the range of 10.67–16.70 kcal/mol (Table 1). The M052X functional (smd, α=1,2) is excluded from the latter range since it smooths out differences between T2–T4 forms in water. Our results, concerning stability of the tautomeric forms of 5FU, are in agreement with the results demonstrated by Markova et al. [58]. Thus, the T1 tautomer appears to be fundamental (Figure 1) for analysis of the protonated and deprotonated forms of 5FU and is used for further consideration.

### 3.3. Deprotonated and Protonated Forms of 5FU in the Gas Phase

The relative energies for the DN1 and DN3 single-deprotonated, as well as for the PO2 and PO4 protonated forms of 5FU were calculated at different levels of theory (**1**–**9**, Table 2). 

All of the methods used herein indicate that the DN1 anion is much more stable than the DN3 anion in the gas phase. The difference in stability of DN1 and DN3 is about 10 kcal/mol (Δ*G*_A_). The greater stability of the DN1 anion results from advantageous delocalisation of the negative charge. In fact, the DN1 anion is represented by four resonance structures (Scheme 3), which illustrate delocalisation of the negative charge onto the N1, O2, C5 and O4 atoms. In turn, the DN3 anion is represented by three resonance structures, which illustrate delocalisation of the negative charge onto the N3, O2 and O4 atoms only. Additionally, an inductive effect caused by the fluorine atom should much more effectively delocalise the negative charge on the DN1 anion than on the DN3 anion due to the close proximity of the negative charge on the C5 atom and the fluorine atom in DN1.

To gain a deeper insight into the negative charge delocalisation in DN1 and DN3 anions, the NBO and ESP analyses were carried out at the B3LYP/6-311++G** (**1**) and M062X/6-311++G** (**3**) levels of theory, respectively (Table 3). The results of these analyses confirm that the DN1 anion possesses a better-delocalised negative charge than the DN3 anion since there is less of a negative charge on the N1 atom in the DN1 anion than on the N3 atom in the DN3 anion. For example, the NBO analysis with M062X/6-311++G** method (**3**) shows that the negative charge on the N1 atom in DN1 anion is −0.65680, whereas the negative charge on the N3 atom in the DN3 anion is −0.69441. In both the DN1 and DN3 anions, the negative charge is delocalised onto the O2 (−0.72892 and −0.74066, respectively) and O4 oxygens (−0.70419 and −0.68767, respectively), which is in agreement with the resonance structures (Scheme 3). However, the O2 oxygen takes a more negative charge (from −0.71649 to −0.79926, depending on method) than the O4 oxygen (from −0.67777 to −0.74247, depending on method) in both the DN1 and DN3 anions. Importantly, the presented results confirm that the negative charge is delocalised onto the C5 carbon atom in the DN1 anion, which is not the case in the DN3 anion. Respective charges on the C5 carbon are always smaller (NBO) or even negative (ESP) for the DN1 anion compared with the DN3 anion. For example, the charge of the C5 atom is 0.16543 in DN1, whereas it is 0.26248 in the DN3 anion, according to the NBO analysis with the M062X/6-311++G** method (**3**). A slightly greater negative charge on the fluorine atom in the DN1 anion than in the DN3 anion may be evidence that an inductive effect of the fluorine atom stabilises the former anion. For example, the charge on the fluorine atom in DN1 is −0.37704, whereas it is −0.36861 in the DN3 anion, according to the NBO analysis with the M062X/6-311++G** method (**3**).

**Figure 2:**
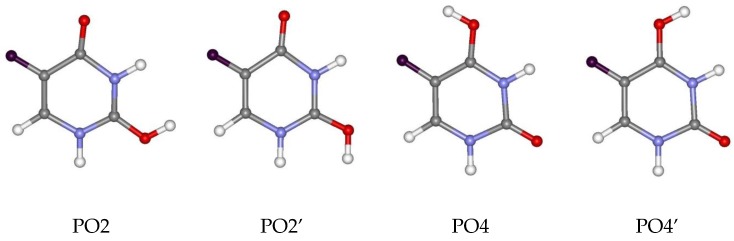
Optimised protonated forms of 5FU with different hydrogen orientations.

In case of protonation of 5FU, two orientations of the hydrogen atom are considered for both protonated forms (Figure 2). These are assigned: PO2 (protonated at the O2 oxygen with hydrogen directed towards the N3-H site), PO2’ (protonated at the O2 oxygen with hydrogen directed towards the N1-H site), PO4 (protonated at the O4 oxygen with hydrogen directed towards the F atom) and PO4’ (protonated at the O4 oxygen with hydrogen directed towards the N3-H site). Irrespective of the hydrogen orientation, the O4 oxygen atom shows a greater affinity to proton than the O2 oxygen atom in a gas phase. This is demonstrated by the calculated relative energies of the cationic forms (Table 2). According to all of the methods used, the PO4 form is the most stable. Changing the hydrogen orientation, PO4→PO4’, increases the energy by 4.91–5.44 kcal/mol, depending on the method used. Greater stability of the PO4 form over the PO4’ form probably results from the electrostatic interactions between the electronegative fluorine atom and the hydrogen atom. Nevertheless, the PO4’ form is still more stable than both forms protonated at the O2 oxygen (PO2 and PO2’). The Δ*G*_C_ value for the PO2 protonated form is in the range of 6.96 to 7.91 kcal/mol, whereas for the PO2’ protonated form is in the range of 7.92 to 8.95 kcal/mol.

The fact that the O4 oxygen atom in 5FU is preferentially protonated, regardless of the hydrogen orientation, is due to the more advantageous delocalisation of the positive charge in the PO4 cation than in the PO2 cation. As shown in Scheme 4, protonation of the O4 oxygen in the T1 tautomeric form allows positive charge to be delocalised onto the five atoms (O4, C4, N3, C6 and N1). In the case of the O2 oxygen protonation in the T1 tautomeric form, delocalisation is reduced to the four atoms (O2, C2, N1 and N3). To verify the charge distribution in the protonated forms of 5FU, the NBO and ESP analyses were carried out at the B3LYP/6-311++G** (**1**) and M062X/6-311++G** (**3**) levels of theory, respectively (Table 4). The more stable PO4 and PO2 structures were used for these studies. The results of the performed calculations are discussed below as an example of the NBO analysis with the M062X/6-311++G** method (**3**).

Protonation of the O2 oxygen atom in 5FU results in the PO2 cationic form with positive charge delocalisation involving the C2=O2 carbonyl group and the neighbouring N1 and N3 nitrogen atoms (Scheme 4). Therefore, the positive charge on the C2 carbon atom (0.85539, Table 4) is much bigger than the positive charge on the C4 carbon atom in the PO2 form (0.63262). However, protonation of the O4 oxygen in 5FU, which gives the PO4 form, does not cause a bigger accumulation of the positive charge on the C4 carbon atom (0.64624) than the C2 carbon atom (0.82381). This indicates that the positive charge in the cationic PO4 form is delocalised in the larger area. As illustrated in Scheme 4, the C6 carbon atom takes place in the delocalisation of the positive charge in the PO4 form, which is not the case in the PO2 form. Indeed, comparison of the C6 carbon atom charges in the PO4 (0.12090) and PO2 (−0.01960) forms confirms that the positive charge in the former cation is partially located at the C6 carbon atom. This is also reflected in the comparison of the H_C_ hydrogen positive charge, which is slightly bigger in the PO4 (0.26711) than in the PO2 (0.26595) form.

### 3.4. Deprotonated and Protonated Forms of 5FU in Water

The exchange of the gas phase by water solution distinctly impacts stabilities of the anionic and cationic forms of 5FU. This is because in a gas phase, the energy of charge delocalisation solely influences the anionic and cationic forms stabilities, whereas in water, the energy of their hydration must be additionally taken into account. Typically, the ions with a better delocalised charge possess smaller dipole moment than those of a worse delocalised charge. Therefore, the latter are better hydrated. Thus, there is a competition between two factors stabilizing the anionic and cationic forms of 5FU in the aqueous phase.

The presented calculations, irrespective of the method used, indicate that the DN1 deprotonated form of 5FU is still more stable than the DN3 deprotonated form in water (Table 5). However, the energy difference between the DN1 and DN3 forms is significantly reduced in an aqueous phase compared with a gas phase and is in the range of 0.56 to 4.10 kcal/mol, depending on the method used (2.08 kcal/mol on average). Such a result differs from that result reported by Jang et al., who found the DN3 anion of 5FU to be 2.44 kcal/mol more stable than the DN1 anion in water [19]. 

The differences between energy of the protonated forms of 5FU are also significantly reduced in an aqueous phase compared with a gas phase due to the competition between charge delocalisation and hydration. However, the PO4 cation remains the most stable protonated form both in the gas and aqueous phases. The difference between the PO4 form and the second most stable form in water is in a range of 0.37 to 2.65 kcal/mol (Table 5), depending on the method used (1.43 kcal/mol on average). The remaining protonated forms do not noticeably differ in terms of the relative energies and the order of their stability in water depends on the method used.

### 3.5. Calculations of the pK_a_ Values

**Scheme 5:**
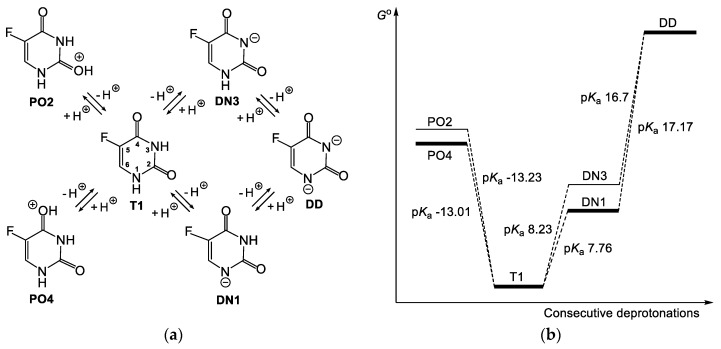
Protonated (PO2, PO4), singly deprotonated (DN1, DN3) and double-deprotonated (DD) forms of 5FU (**a**); scheme of the energy changes related to the acid equilibria of 5FU and respective p*K*_a_ values calculated using the **3cD** methodology (**b**).

Based on the Gibbs free energies obtained with different DFT methods, the p*K*_a_ values for the all possible acidic equilibria concerning the T1 tautomeric form of 5FU (Scheme 5a) were calculated. Two methodologies of p*K*_a_ calculations, direct (**D**) and relative (**R**), were applied. The results of our investigations (Table 6) show that the calculated p*K*_a_ values, related to the specific acid equilibrium, strongly depend on the DFT method (**1**–**8**), solvation model (**a**–**c**) and thermodynamic cycle (**D** or **R**). These show how difficult it is to find the most applicable DFT method for the accurate p*K*_a_ calculation of such a multi-faceted compound as 5FU. It does not change the fact that the calculated p*K*_a_ values for deprotonation of the N1 nitrogen in the T1 tautomer of 5FU (T1⇌DN1) are, generally, slightly lower than the p*K*_a_ values for deprotonation of the N3 nitrogen (T1⇌DN3). This is due to the above-demonstrated better stabilization and lower energy of the DN1 deprotonated form compared with the DN3 deprotonated form in water (Table 5). The p*K*_a_ values for the T1⇌DN1 acid equilibrium in methodology **D** are in the range of 6.65 to 11.49 (9.56 on average), whereas in methodology **R** in the range of 1.75 to 8.25. (5.25 on average). In turn, the p*K*_a_ values for the T1⇌DN3 acid equilibrium in methodology **D** are in the range of 6.64 to 13.25 (10.21 on average) whereas in methodology **R**, they are in the range of 2.22 to 8.92 (6.60 on average). Generally, methodology **D** provides higher p*K*_a_ values than methodology **R**.

There are seven methodologies among those tested herein, which provide the p*K*_a_ T1→A value of 5FU in the range of 7.51–8.20, which is close to the experimentally measured value of 7.93 [6] or 8.05 [7] (Table 6). Four of them, the **3cD**, **5bR**, **6bR** and **7bD** methods, give the clearly lower p*K*_a_ values for deprotonation of the N1 nitrogen (7.76, 7.51, 8.18 and 8.15, respectively) than for deprotonation of the N3 nitrogen (8.23, 8.75, 8.92 and 8.80, respectively). Three remaining methods, namely **5cR**, **6cR**, and **8bD** provide almost the same p*K*_a_ T1→A value for deprotonations of the N1 and N3 nitrogens, indicating that both nitrogens are equally likely to be deprotonated in water. Such results are due to small differences in stability of the DN1 and DN3 anions, calculated with these methodologies (Table 5). Taking into account the averaged results of all methodologies used here, the lower p*K*_a_ value is provided for the T1⇌DN1 equilibrium than for the T1⇌DN3 equilibrium (∆p*K*_a_ = 0.65 and 1.35 for methodology **D** and **R**, respectively).

Since the DN1 anion is more stable than the DN3 anion (Table 5), the second deprotonation related to the DN3⇌DD equilibrium is expected to occur more easily than deprotonation related to the DN1⇌DD equilibrium (Scheme 5b). Therefore, the calculated p*K*_a_ values for the DN1⇌DD equilibrium are generally higher than those calculated p*K*_a_ values for the DN3⇌DD equilibrium (Table 6). The former ones are in a range of 16.92 to 29.08 (21.10 on average), whereas the latter in the range of 16.70 to 26.90 (20.44 on average) when methodology **D** is used. In methodology **R**, these values are in the range of 11.19 to 23.55 (18.19 on average) and 10.72 to 21.37 (17.28 on average), respectively. It does not change the fact that the second p*K*_a_ value of 5FU must be related to the DN1⇌DD equilibrium if the first p*K*_a_ value of 5FU is related to the DN1 anion.

Looking at the **3cD** method, which provides the first p*K*_a_ value of 5FU (7.76) in accordance with the experimental value (7.93 [6] or 8.05 [7]), we may state that the second p*K*_a_ value of 5FU is 17.17, and is related to the DN1⇌DD equilibrium. Six other above-mentioned methodologies, which provide the first p*K*_a_ value of 5FU close to the experimental p*K*_a_ value, place the second p*K*_a_ value in the range of 17.24 to 18.93. The second p*K*_a_ value of 5FU in the range of 17.24 to 18.93 seems to be reasonable, given that the dianion needs to be created. It must be added that the available literature data on the second p*K*_a_ value of 5FU differ from these reported herein and are 9.0 [22] or 13.0 [26]. On the other hand, a p*K*_a_ value higher than 14 is not measured in water, which might explain why the data on the second p*K*_a_ of 5FU are limited. Importantly, based on the second p*K*_a_ values of 5FU calculated here, we may state that the dianionic form of 5FU is strongly limited in aqueous media, even if it is the alkaline aqueous solution.

It was demonstrated above that the O4 carbonyl oxygen is more easily protonated than the O2 carbonyl oxygen. Therefore, the O2 protonated 5FU is expected to be a stronger acid than the O4 protonated 5FU. In fact, the calculated p*K*_a_ values for the PO4⇌T1 equilibrium are higher than the calculated p*K*_a_ values for the PO2⇌T1 equilibrium, irrespective of the methodology used. The p*K*_a_ values for PO4⇌T1 equilibrium are in the range from −20.50 to 8.24 (−6.43 on average), whereas for the PO2⇌T1 equilibrium, they are in the range from −22.23 to 7.63 (−7.20 on average) when methodology **D** is used. In methodology **R**, these values are in the range from −27.99 to 4.96 (−12.40 on average) and from −29.72 to 4.34 (−13.42 on average), respectively. Particular p*K*_a_ values concerning the protonated forms of 5FU differ widely depending on the calculation method used. In our opinion, some of them are underestimated, these are the p*K*_a_ values obtained with the **1aD**, **3aD** and **1aR**–**4aR** methods, which quite often are below value −20. On the other hand, some are overestimated, these are the p*K*_a_ values obtained with the **5b**, **5c**, **6b** and **6c** methods, for both the **R** and **D** thermodynamic cycles, which are found within the range of 3.80–8.24. Such a range of the p*K*_a_ values for the protonated form of 5FU is hard to accept. Thus, the WB97XD methods (**5b**,**c** and **6b**,**c**), which provide very accurate p*K*_a_ values for deprotonation of the neutral 5FU when the relative methodology (**R**) is used, do not work in the case of p*K*_a_ calculations of the cationic form of 5FU. Three other effective methods in deprotonation calculations of the neutral 5FU, namely the **3cD**, **7bD** and **8bD**, provide p*K*_a_ values of −13.01, −11.72 and −12.71, respectively, for the PO4⇌T1 equilibrium, and p*K*_a_ values of −13.23, −12.17 and −13.02, respectively, for the PO2⇌T1 equilibrium. It is difficult to verify these values because, to our knowledge, the p*K*_a_ value of the protonated 5FU has not been measured. This is due to the fact that the protonated 5FU must be a strong acid with a p*K*_a_ value that is impossible to measure in a water solution. Importantly, based on the p*K*_a_ values of the protonated 5FU calculated here, we may state that both the protonated forms of 5FU, PO2 and PO4 are strongly limited in aqueous media, even if it is the acidic aqueous solution. 

## 4. Conclusions

5-Fluorouracil, a drug with two potential sites of protonation and two potential sites of deprotonation, occurs in four tautomeric forms, among which the 2,4-dioxo tautomer (T1) is evidently the most stable. The protonated and deprotonated forms of 5FU, respectively, create systems of connected equilibriums with the tautomeric forms of 5FU. The gas-phase calculations indicate that the O4 carbonyl oxygen of 5FU is much more easily protonated than the O2 carbonyl oxygen, and the N1 nitrogen is much more easily deprotonated than the N3 nitrogen. The demonstrated preferences result from advantageous charge delocalisation of the respective ions. In an aqueous phase, stability differences between respective protonated and deprotonated forms are significantly diminished due to solvation. However, the O4 oxygen is shown to be slightly more easily protonated than the O2 oxygen and the N1 nitrogen is slightly more easily deprotonated than the N3 nitrogen. Calculations of the p*K*_a_ of protonated, neutral and singly deprotonated 5FU provided wide ranges of respective values, which strongly depend on the method used. It shows how difficult it is to find the most applicable DFT method for accurate p*K*_a_ calculation of such a multi-faceted compound like 5FU. Importantly, all the obtained p*K*_a_ values are in line with the calculated stabilities of the protonated and deprotonated forms of 5FU in the aqueous phase. The M062X/6-311++G**, M052X/6-311++G** and M052X/6-31+G** methods with SMD solvation models and direct thermodynamic cycles, namely **3cD**, **7bD** and **8bD**, provide the most accurate p*K*_a_ values for all of the considered acid equilibriums. The **3cD** and **7bD** methods clearly indicate that the N1 nitrogen is more willingly deprotonated than the N3 nitrogen, which is in accordance with the averaged results from all the methods tested herein. In turn, the **8bD** method indicates that both nitrogens are equally likely to be deprotonated in water. Based on the **3cD** and **7bD** methods, the acidic equilibriums concerning 5FU are expected to be shaped as follows: PO4⇌T1⇌DN1⇌DD (Scheme 5) with the following p*K*_a_ values: −13.01 (**3cD**) or −11.72 (**7bD**) for the PO4 form, 7.76 (**3cD**) or 8.15 (**7bD**) for the T1 form and 17.17 (**3cD**) or 19.00 (**7bD**) for the DN1 form. However, it cannot be missed that a few methods presented here, being in the minority, do not exactly fit into this scheme. These indicate that the O2 and O4 atoms of 5FU are equally willingly protonated and N1 and N3 atoms are equally willingly deprotonated. It does not change the fact that 5FU does not exist in the protonated and double-deprotonated forms in a pH range of 0–14, which is characteristic of water. The neutral form of 5FU dominates below pH 8 and the N1 deprotonated form of 5FU dominates above pH 8.

## Figures and Tables

**Table 1:** Calculated relative energies for the T1–T4 tautomeric forms of 5FU (Δ*G*_T_, kcal/mol) in the gas phase and in water.

Gas Phase			Water			
Method ^a^	Tautomer	Δ*G*_T_	Method	Solvation Model (α) ^b^	Tautomer	Δ*G*_T_
B3LYP/	T1	0.00	B3LYP/	pcm (1,2)	T1	0.00
6-311++G**	T2	9.70	6-311++G**	(**1a**)	T2	11.68
(**1**)	T3	13.00			T3	12.50
	T4	12.71			T4	16.70
B3LYP/	T1	0.00	B3LYP/	pcm (1,2)	T1	0.00
aug-cc-pVDZ	T2	8.83	aug-cc-pVDZ	(**2a**)	T2	10.86
(**2**)	T3	11.83			T3	11.49
	T4	10.71			T4	14.98
M062X/	T1	0.00	M062X/	pcm (1,2)	T1	0.00
6-311++G**	T2	8.25	6-311++G**	(**3a**)	T2	10.16
(**3**)	T3	12.13			T3	11.39
	T4	9.71			T4	13.46
M062X/	T1	0.00	M062X/	pcm (1,2)	T1	0.00
aug-cc-pVDZ	T2	7.32	aug-cc-pVDZ	(**4a**)	T2	9.27
(**4**)	T3	10.88			T3	10.32
	T4	7.64			T4	11.63
WB97XD/	T1	0.00	WB97XD/	smd (1,2)	T1	0.00
6-31+G**	T2	9.85	6-31+G**	(**5b**)	T2	8.53
(**5**)	T3	13.19			T3	8.93
	T4	12.25			T4	10.67
WB97XD/	T1	0.00	WB97XD/	smd (1,2)	T1	0.00
6-311++G**	T2	10.38	6-311++G**	(**6b**)	T2	8.99
(**6**)	T3	13.82			T3	9.60
	T4	13.47			T4	11.71
M052X/	T1	0.00	M052X/	smd (1,2)	T1	0.00
6-31+G**	T2	7.67	6-31+G**	(**7b**)	T2	6.48
(**7**)	T3	11.40			T3	7.09
	T4	8.20			T4	6.89
M052X/	T1	0.00	M052X/	smd (1,2)	T1	0.00
6-311++G**	T2	8.19	6-311++G**	(**8b**)	T2	6.95
(**8**)	T3	12.09			T3	7.81
	T4	9.48			T4	7.99
CBS-QB3	T1	0.00				
(**9**)	T2	10.01				
	T3	13.33				
	T4	13.19				

^a^ Methods of calculations are assigned by numbers **1**–**9**. ^b^ Solvation models are assigned by letters **a** and **b**.

**Table 2:** Calculated relative energies (kcal/mol) for anionic (Δ*G*_A_) and cationic (Δ*G*_C_) forms of 5FU in the gas phase.

Method ^a^	Anions		Cations	
	Form	Δ*G*_A_	Form	Δ*G*_C_
B3LYP/	DN1	0.00	PO4	0.00
6-311++G**	DN3	10.89	PO4’	4.91
(**1**)			PO2	7.84
			PO2’	8.95
B3LYP/	DN1	0.00	PO4	0.00
aug-cc-pVDZ	DN3	10.80	PO4’	4.76
(**2**)			PO2	7.81
			PO2’	8.82
M062X/	DN1	0.00	PO4	0.00
6-311++G**	DN3	11.48	PO4’	5.06
(**3**)			PO2	7.03
			PO2’	8.09
M062X/	DN1	0.00	PO4	0.00
aug-cc-pVDZ	DN3	10.42	PO4’	4.91
(**4**)			PO2	6.96
			PO2’	7.92
WB97XD/	DN1	0.00	PO4	0.00
6-31+G**	DN3	11.29	PO4’	5.44
(**5**)			PO2	7.91
			PO2’	8.92
WB97XD/	DN1	0.00	PO4	0.00
6-311++G**	DN3	10.96	PO4’	5.28
(**6**)			PO2	7.59
			PO2’	8.56
M052X/	DN1	0.00	PO4	0.00
6-31+G**	DN3	11.11	PO4’	5.31
(**7**)			PO2	7.84
			PO2’	8.85
M052X/	DN1	0.00	PO4	0.00
6-311++G**	DN3	9.93	PO4’	5.24
(**8**)			PO2	7.56
			PO2’	8.61
CBS-QB3	DN1	0.00		
(**9**)	DN3	12.32		

^a^ Methods of calculations are assigned by numbers **1**–**9**.

**Table 3:** The charge distribution in the DN1 and DN3 anions from NBO and ESP analyses.

Atom	B3LYP/6-311++G** (1)				M062X/6-311++G** (3)			
	DN1		DN3		DN1		DN3	
	NBO	ESP	NBO	ESP	NBO	ESP	NBO	ESP
N1	−0.64160	−0.87928	−0.62546	−0.67064	−0.65680	−0.87076	−0.65107	−0.68324
C2	0.75691	1.14084	0.74969	1.06060	0.78306	1.12004	0.78291	1.03724
O2	−0.71649	−0.79926	−0.72704	−0.79611	−0.72892	−0.78939	−0.74066	−0.78536
N3	−0.63861	−0.86443	−0.66903	−0.96868	−0.66353	−0.87699	−0.69441	−0.95796
C4	0.56819	0.82014	0.54955	0.93854	0.59520	0.81466	0.57438	0.91704
O4	−0.69612	−0.71467	−0.67777	−0.74247	−0.70419	−0.70408	−0.68767	−0.72959
C5	0.17966	−0.11270	0.26282	0.02598	0.16543	−0.12635	0.26248	0.02735
C6	0.01039	0.19541	−0.06090	−0.11183	0.01927	0.18140	−0.05630	−0.12355
F	−0.37576	−0.25392	−0.36543	−0.25231	−0.37704	−0.24870	−0.36861	−0.24879
H_C_^a^	0.16596	0.09509	0.18129	0.16590	0.17319	0.11285	0.18983	0.18207
H_N_^a^	0.38746	0.37278	0.38228	0.35103	0.39434	0.38734	0.38911	0.36479

^a^ The subscript indicates an atom on which a respective hydrogen is placed.

**Table 4:** The charge distribution in PO2 and PO4 cations from NBO and ESP analyses.

Atom	B3LYP/6-311++G** (1)				M062X/6-311++G** (3)			
	PO2		PO4		PO2		PO4	
	NBO	ESP	NBO	ESP	NBO	ESP	NBO	ESP
N1	−0.52172	−0.37637	−0.54624	−0.44314	−0.54561	−0.39133	−0.57441	−0.47095
C2	0.82453	0.71708	0.79747	0.72929	0.85539	0.73047	0.82381	0.73340
O2	−0.58743	−0.53626	−0.49825	−0.45452	−0.60298	−0.54483	−0.50598	−0.44923
N3	−0.59010	−0.56479	−0.55623	−0.46815	−0.61740	−0.59039	−0.58116	−0.48926
C4	0.60941	0.63068	0.61677	0.53756	0.63262	0.63635	0.64624	0.55605
O4	−0.45348	−0.40000	−0.56529	−0.45044	−0.45754	−0.39176	−0.58214	−0.46086
C5	0.33519	0.21267	0.24963	0.08462	0.33339	0.20824	0.23501	0.06382
C6	−0.01754	−0.16055	0.10877	0.05337	−0.01960	−0.17216	0.12090	0.06358
F	−0.27028	−0.12250	−0.29963	−0.14171	−0.27896	−0.12694	−0.30502	−0.14267
H_C_^a^	0.25859	0.26240	0.26027	0.23840	0.26595	0.27593	0.26711	0.24881
H_N1_^a^	0.45185	0.42961	0.44978	0.40651	0.44843	0.41084	0.46509	0.43472
H_N3_^a^	0.44031	0.39876	0.45650	0.42280	0.45973	0.43911	0.45746	0.41824
H_O_^a^	0.52067	0.50927	0.52645	0.48541	0.52659	0.51647	0.53309	0.49435

^a^ The subscript indicates an atom on which a respective hydrogen is placed.

**Table 5:** Calculated relative energies (kcal/mol) for anionic (Δ*G*_A_) and cationic (Δ*G*_C_) forms of 5FU in water.

Method	Solvation Model (α) ^a^	Anions		Cations	
		Form	Δ*G*_A_	Form	Δ*G*_C_
B3LYP/	pcm (1,2)	DN1	0.00	PO4	0.00
6-311++G**	(**1a**)	DN3	4.10	PO4’	2.59
**1**				PO2’	3.13
				PO2	3.73
	smd (1,0)	DN1	0.00	PO4	0.00
	(**1c**)	DN3	1.39	PO2’	0.37
				PO2	0.99
B3LYP/	pcm (1,2)	DN1	0.00	PO4	0.00
aug-cc-pVDZ	(**2a**)	DN3	3.86	PO4’	2.64
**2**				PO2’	3.53
				PO2	3.82
M062X/	pcm (1,2)	DN1	0.00	PO4	0.00
6-311++G**	(**3a**)	DN3	3.63	PO2’	2.51
**3**				PO4’	2.58
				PO2	2.90
	smd (1,0)	DN1	0.00	PO4	0.00
	(**3c**)	DN3	0.91	PO2	1.00
M062X/	pcm (1,2)	DN1	0.00	PO4	0.00
aug-cc-pVDZ	(**4a**)	DN3	3.38	PO4’	2.65
**4**				PO2’	2.75
				PO2	2.95
WB97XD/	smd (1,2)	DN1	0.00	PO4	0.00
6-31+G**	(**5b**)	DN3	1.93	PO4’	0.80
**5**				PO2’	0.94
				PO2	0.97
	smd (1,0)	DN1	0.00	PO4	0.00
	(**5c**)	DN3	0.86	PO2	1.46
WB97XD/	smd (1,2)	DN1	0.00	PO4	0.00
6-311++G**	(**6b**)	DN3	2.03	PO4’	0.71
**6**				PO2	0.78
				PO2^’^	0.79
	smd (1,0)	DN1	0.00	PO4	0.00
	(**6c**)	DN3	0.98	PO2	1.30
M052X/	smd (1,2)	DN1	0.00	PO4	0.00
6-31+G**	(**7b**)	DN3	1.63	PO2’	0.76
**7**				PO4’	0.77
				PO2	0.79
	smd (1,0)	DN1	0.00	PO4	0.00
	(**7c**)	DN3	0.56	PO2	1.31
M052X/	smd (1,2)	DN1	0.00	PO4	0.00
6-311++G**	(**8b**)	DN3	1.81	PO2	0.56
**8**				PO2’	0.61
				PO4’	0.66

^a^ Methods of calculations are assigned by numbers **1**–**8**, the solvation models are assigned by letters **a**–**c**.

**Table 6:** The p*K*_a_ values for acidic equilibria of 5FU concerned the T1 tautomeric form.

Method	Solvation Model (α) ^a^	Anion	p*K*_a_ T1→A ^b^		p*K*_a_ A→DD		Cation	p*K*_a_ C ^c^→T1	
		A	met. D	met. R	met. D	met. R	C	met. D	met. R
B3LYP/	pcm (1,2)	DN1	11.07	5.48	29.08	23.55	PO4	−19.09	−24.32
6-311++G**	**1a**	DN3	13.25	7.67	26.90	21.37	PO2	−21.38	−27.07
	smd (1,0)	DN1	9.22	4.80	18.97	14.58	PO4	−12.25	−16.73
	**1c**	DN3	9.33	4.91	18.86	14.47	PO2	−12.46	−16.94
B3LYP	pcm (1,2)	DN1		5.29		23.36	PO4		−23.23
aug-cc-pVDZ	**2a**	DN3		7.75		20.90	PO2		−26.23
M062X	pcm (1,2)	DN1	9.98	2.59	27.92	20.58	PO4	−20.50	−27.99
6-311++G**	**3a**	DN3	12.55	5.17	25.35	18.01	PO2	−22.23	−29.72
	smd (1,0)	DN1	7.76	1.75	17.17	11.19	PO4	−13.01	−19.08
	**3c**	DN3	8.23	2.22	16.70	10.72	PO2	−13.23	−19.31
M062X	pcm (1,2)	DN1		2.09		20.10	PO4		−27.28
aug-cc-pVDZ	**4a**	DN3		4.25		17.94	PO2		−29.13
WB97XD	smd (1,2)	DN1	11.17	7.51	21.95	18.71	PO4	8.24	4.96
6-31+G**	**5b**	DN3	12.02	8.75	21.10	17.86	PO2	7.63	4.34
	smd (1,0)	DN1	10.79	8.03	19.98	17.25	PO4	7.65	4.88
	**5c**	DN3	10.80	8.04	19.97	17.24	PO2	7.03	4.25
WB97XD	smd (1,2)	DN1	11.49	8.18	22.21	18.93	PO4	7.63	4.30
6-311++G**	**6b**	DN3	12.24	8.92	21.47	18.18	PO2	7.16	3.83
	smd (1,0)	DN1	11.11	8.25	20.24	17.44	PO4	7.14	4.30
	**6c**	DN3	11.02	8.20	20.32	17.53	PO2	6.64	3.80
M052X	smd (1,2)	DN1	8.15	6.40	19.00	17.29	PO4	−11.72	−13.53
6-31+G**	**7b**	DN3	8.80	7.05	18.35	16.64	PO2	−12.17	−13.98
	smd (1,0)	DN1	6.65	6.56	16.92	15.77	PO4	−12.14	−13.66
	**7c**	DN3	6.44	6.34	17.13	15.98	PO2	−13.19	−14.11
M052X	smd (1,2)	DN1	7.77	6.66	18.64	17.67	PO4	−12.71	−13.88
6-311++G**	**8b**	DN3	7.66	6.55	18.75	17.78	PO2	−13.02	−14.20

^a^ Methods of calculations are assigned by numbers **1**–**8**, the solvation models by letters **a**–**c**. ^b^ “A” means anionic forms: DN1 or DN3. ^c^ “C” means cationic forms: PO2 or PO4.
